# Psychotropic drug prescription rates in primary care for people with dementia from recorded diagnosis onwards

**DOI:** 10.1002/gps.5442

**Published:** 2020-10-16

**Authors:** Karlijn J. Joling, Maud ten Koppel, Hein P.J. van Hout, Bregje D. Onwuteaka‐Philipsen, Anneke L. Francke, Robert A. Verheij, Jos W.R. Twisk, Rob J. van Marum

**Affiliations:** ^1^ Department of General Practice and Elderly Care Medicine Amsterdam UMC Vrije Universiteit Amsterdam Amsterdam Public Health Research Institute Amsterdam The Netherlands; ^2^ Department of Public and Occupational Health, Expertise Center for Palliative Care Amsterdam UMC Vrije Universiteit Amsterdam Amsterdam Public Health Research Institute Amsterdam The Netherlands; ^3^ Nivel. Netherlands Institute for Health Services Research Utrecht The Netherlands; ^4^ Department of Epidemiology and Biostatistics Amsterdam UMC Vrije Universiteit Amsterdam Amsterdam Public Health Research Institute Amsterdam The Netherlands; ^5^ Geriatric Department Jeroen Bosch Hospital ‘s‐Hertogenbosch The Netherlands

**Keywords:** dementia, electronic health records, primary care, psychotropic drug prescriptions

## Abstract

**Background:**

Psychotropic drugs are frequently prescribed to people with dementia in nursing homes although severe adverse events and side effects are common. Less is known about the prevalence and types of psychotropic drug prescription in primary care for people with dementia.

**Objective:**

This study examined the prevalence of psychotropic drug prescriptions in primary care among persons with dementia from the year of diagnosis onwards.

**Methods:**

A longitudinal observational study using electronic health record (EHR) data was conducted. People with dementia were selected from EHR data of 451 general practices in the Netherlands. Age and gender‐adjusted psychotropic drug prescription rates were calculated per 1000 person‐years from the year the dementia diagnosis was first recorded in general practice up to 8 years after diagnosis.

**Results:**

Data of 15,687 patients were analyzed. The prescription rate of psychotropic drugs (not including antidementia drugs) was 420 per 1000 person‐years (95% CI 409; 431) in the first year after the recorded dementia diagnosis, which increased to 801 per 1000 person‐years (95% CI 649; 989) in the eighth year. The most frequently prescribed drugs were antidepressants, antipsychotics, and antidementia drugs, followed by anxiolytics, hypnotics, and antiepileptics.

**Conclusions:**

After a dementia diagnosis is recorded in general practice, the prevalence of psychotropic drug prescriptions is substantial and increases steadily during the disease trajectory of persons with dementia. Although the (in)appropriateness of prescribing was not assessed, these insights may stimulate primary care clinicians to (re)consider their prescription policy of psychotropics for people with dementia more carefully.

## 1 INTRODUCTION

The majority of community‐dwelling people with dementia experience one or more neuropsychiatric symptoms, such as delusion, agitation, or apathy.^1^ The occurrence of these symptoms increases throughout the disease trajectory.^1^, ^2^ As the first choice for treatment of neuropsychiatric symptoms in people with dementia, clinical guidelines recommend nonpharmacological approaches.^3^, ^4^, ^5^, ^6^ Pharmacological treatment with psychotropic drugs should only be used when there is no response to nonpharmacological approaches and when symptoms are burdensome for the patient or his environment. Many psychotropic drugs have shown limited efficacy for the treatment of neuropsychiatric symptoms^7^, ^8^ and the occurrence of side effects and adverse events, such as worsening of cognitive functioning, falls, and sedation, are common in people with dementia.^3^, ^4^, ^5^, ^6^, ^9^ It is therefore recommended that clinicians exercise restraint in prescribing psychotropics to people with dementia and use alternative strategies where possible.

Nevertheless, psychotropic drugs are frequently prescribed to people with dementia in nursing homes, with worldwide estimates of 66%–79%,^9^, ^10^, ^11^ and residents are likely to receive these drugs continuously.^12^ However, sound estimates of psychotropic drug prescribing in primary care for people with dementia are limited. Only two studies with large representative samples investigated psychotropic drug prescription rates over longer periods of time.^13^, ^14^ In Finland, 37% of community‐dwelling people with dementia were estimated to use psychotropics at the time of diagnosis, increasing to 50% 4 years after diagnosis.^14^ In the United Kingdom, a mean prevalence of 13% for antipsychotics, 22% for antidepressants, and 10% and 5% for hypnotics and anxiolytics respectively, was reported in people with dementia in general practice at the time of diagnosis.^13^ The few other studies conducted among people with dementia in the community were cross sectional, used small and selective study samples with unclear disease onset and/or reported only the prevalence of a specific psychotropic drug.^15^, ^16^, ^17^, ^18^, ^19^, ^20^

**Key Points**

- Psychotropic drug prescriptions in primary care were substantial in people with dementia
- These prescription rates increased significantly during the disease trajectory
- Antidepressants and antipsychotics were most often prescribed
- Results may stimulate primary care clinicians to (re)consider their prescription policy

Because the risk of developing behavioral and psychological symptoms increases during the progression of dementia,^1^, ^2^ it is likely that the use of psychotropic drugs will also increase over the course of the disease. Providing insight into prescription rates of psychotropic drugs among people with dementia in primary care across the disease trajectory can help clinicians to become more aware of their prescription practices. This is important to reduce inappropriate prescribing and associated adverse effects.

This study examined the prevalence of the prescription of different types of psychotropic drugs in primary care during the disease trajectory of people with dementia from the year the diagnosis was first recorded in general practice up to the eighth year after the diagnosis.

## 2 MATERIALS AND METHODS

### 2.1 Study design

Longitudinal observational study using electronic health record (EHR) data to gain insight into the prevalence of psychotropic drug prescriptions in people with dementia.

### 2.2 Data sources

Data from the NIVEL Primary Care Database (NIVEL‐PCD) were used, covering the period 2008–2015. NIVEL‐PCD includes continuous routine EHR data, and for the purpose of this study, data from 451 general practices in the Netherlands were included (https://www.nivel.nl/en/nivel-primary-care-database). These general practices are representative of Dutch general practices regarding their patients’ gender and age distribution, the practice size and geographical distribution, and cover approximately 10% of the Dutch population. For this study, we used data on prescriptions and diagnoses. Prescriptions are coded according to the Anatomical Therapeutic Chemical (ATC) Classification System.^21^ Diagnoses are coded according to International Classification of Primary Care (ICPC‐1)^22^ and grouped into disease episodes.^23^ General Practitioners (GPs) receive feedback on the quality of recording and are supported in coding.^24^ Additionally, during 2012 and 2013 a part of GPs' reimbursement was based on the quality of recording.^25^

Data from administrative data sources made available for research by Statistics Netherlands Centraal Bureau voor de Statistiek (CBS) were used to derive sociodemographic data and date of death. Date of death originated from the Municipal Personal Records Database, which includes all persons residing in the Netherlands. Sociodemographic information was derived from the national population registry managed by Statistics Netherlands. Linkage of these administrative data with the EHR data of GPs was necessary because follow‐up of patients ended either on the date of leaving the general practice or on the date of death.

### 2.3 Data linkage

NIVEL‐PCD data were pseudonymized at source and transferred to Statistics Netherlands which performed the linkage. Pseudonyms were based on the citizen service number, or a combination of birth date, gender, and zip code.

### 2.4 Study sample

The study sample consisted of patients born in or before 1965, with a first diagnostic code for dementia (ICPC code P70) recorded in the EHR system between 2008 and 2015. We excluded persons with a dementia diagnosis and Down syndrome (ICPC code A90.01), since these persons have different care trajectories and probably also medication prescriptions. Additionally, we excluded persons whose dementia diagnosis was first recorded on or after their date of death, on the date they left the practice or at the end of the data extraction period.

In the Netherlands, dementia is a clinical diagnosis that can be made by GPs or through referral to a medical specialist. When the diagnosis is made by a medical specialist, it has to be copied into the GPs EHR system. In 2016, about 58% of all incident cases of dementia were diagnosed in a memory clinic.^26^

### 2.5 Prevalence of psychotropic drug prescriptions

Psychotropic drugs were divided into the following categories, based on the ATC codes recorded in the GPs EHR system: anxiolytics, antipsychotics, antidepressants, hypnotics, antiepileptics, and antidementia drugs. These categories were further divided into subcategories (Table S1). The prescription rates of any psychotropic drugs included anxiolytic, antipsychotic, antidepressant, hypnotic, and antiepileptic prescriptions. Antidementia drug prescription rates were presented separately as these drugs do not have adverse effects similar to many of the drugs for psychiatric conditions and are prescribed for different reasons.

### 2.6 Sociodemographic and clinical characteristics

Sociodemographics included gender, age, cohabitation (vs. living alone), and migration background. Migration background was categorized according to the classification of Statistics Netherlands into native Dutch, Western, Surinamese/Antillean/Aruban, Moroccan/Turkish, or another non‐Western background. A frailty index was based on a list of 36 predefined health deficits, including ICPC codes of diseases and symptoms, and one deficit “polypharmacy”, as previously described by Drubbel et al.^27^ Calculation of the proportion of deficits resulted in a Frailty Index score (between 0 and 1). Frailty scores were classified into the categories nonfrail, prefrail, and frail (Table 1), based on previous studies (e.g., ^28^, ^29^).

**TABLE 1:** Sociodemographic and clinical characteristics of the study sample (*n* = 15,687)

	*N*	*%*
**Female**	9941	63.4
**Age, mean (SD** ^a^ **)**	81.2	8.1
Under 65	639	4.1
65–74	2463	15.7
75–84	7173	45.7
85 and above	5420	34.6
**Cohabiting**	9173	58.5
Missing	12	0.1
**Migration background**		
Native Dutch	13,712	87.4
Western	1537	9.8
Surinamese/Antillean/Aruban	181	1.2
Moroccan/Turkish	175	1.1
Other non‐Western	82	0.5
**Frailty index (FI, 0‐1),** median (range)	0.11	0.5
mean (SD^a^)	0.14	0.1
Non frail (FI ≤ 0.08)	2757	17.6
Pre‐frail (0.08 > FI > 0.25)	11,660	74.3
Frail (FI ≥ 0.25)	1270	8.1

^a^ Standard deviation.

### 2.7 Ethics

This study was approved by the Medical Ethical Committee of the VU University Medical Center and conducted according to the governance code of Nivel‐PCD (NZR‐00315.063). Data were processed in accordance with national and EU regulations.

### 2.8 Analyses

Psychotropic drug prescription rates were calculated per 1000 person‐years, that is the number of people with a prescription divided by the number of person‐years, multiplied by 1000. The number of people with a prescription was calculated per year for the period between the date that the dementia diagnosis was first recorded in the EHR and the end of the study period (either date of death, date of leaving the GP practice, or end of the observation period). Psychotropic drug prescription rates were calculated with Poisson regression analyses, as Poisson regression calculates event counts during a certain time interval and can take into account different length of follow‐up of patients and incorporate possible confounders. All prescription rates were adjusted for age and gender. Analyses were performed using SPSS, version 22.

## 3 RESULTS

### 3.1 Study sample

**FIGURE 1:**
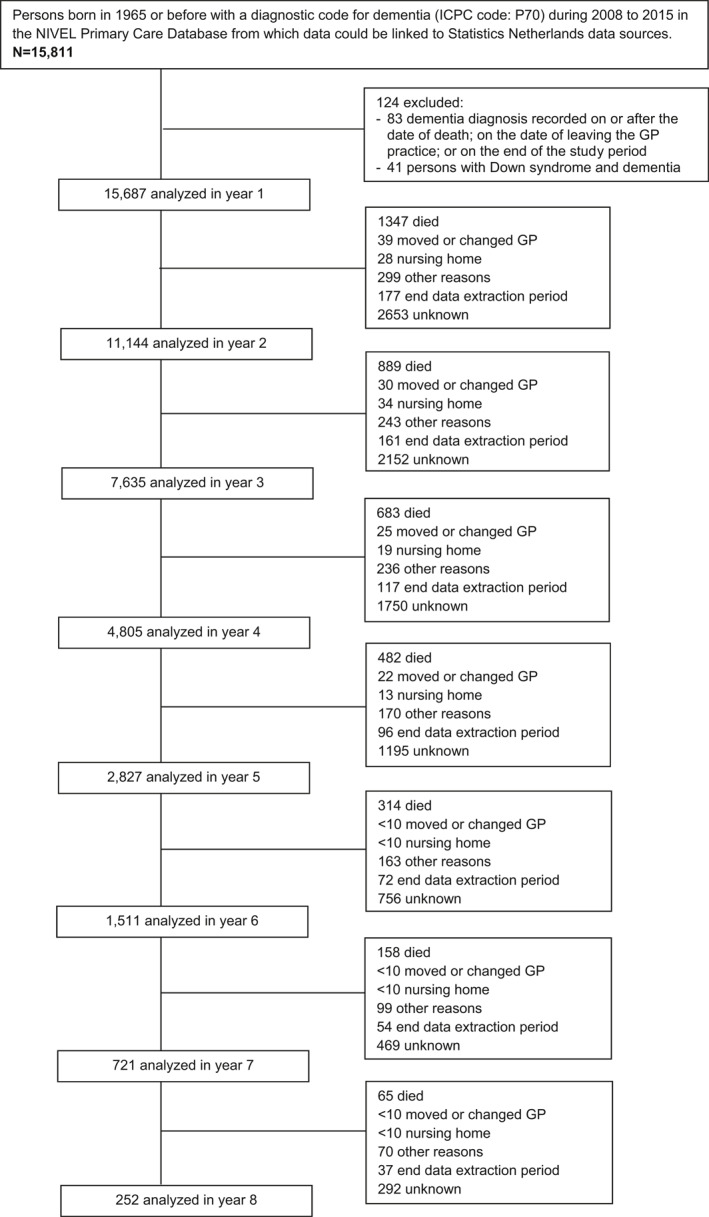
Flow diagram of the study sample

We identified 15,811 persons with a dementia diagnosis in the GPs EHRs between 1 January 2008 and 31 December 2015 in the linked dataset. A total of 15,687 persons with a dementia diagnosis met all inclusion criteria and were included in the analyses (Figure 1). The median follow‐up time in the study was 1.9 years (IQR 0.9–3.4). Sixty‐three percent of the persons with dementia were female and the mean age was 81 years (Table 1). Fifty‐nine percent of the persons with dementia lived together with another person. Most people (87%) had a native Dutch background. Over 80% were pre‐frail or frail.

### 3.2 Psychotropic drug prescription rates over the disease course

**FIGURE 2:**
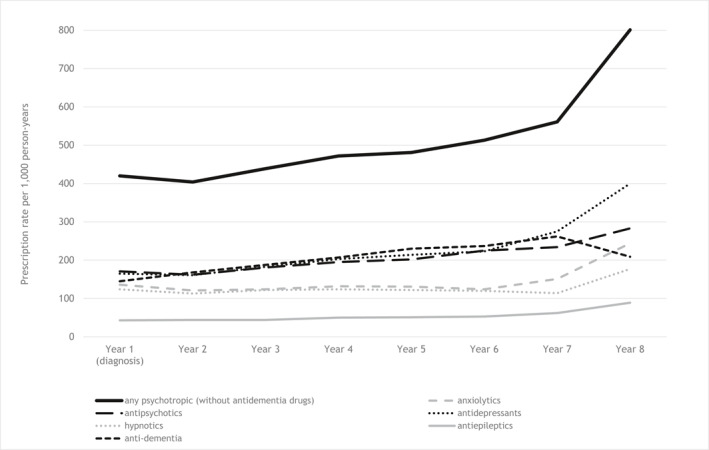
Prevalence of any psychotropic drug prescription and of categories of psychotropic drug prescriptions from the year of recorded dementia diagnosis onwards

Table 2 and Figure 2 show the prescription rates of psychotropic drugs per 1000 person‐years for each year after the dementia diagnosis was first recorded. In the first year, the prescription rate of any psychotropic drug (i.e., anxiolytic, antipsychotic, antidepressant, hypnotic, and/or antiepileptic prescriptions) was 420/1000 person‐years, which steadily and significantly increased to 561/1000 person‐years in the seventh year and rose to 801/1000 person‐years in the 8th year.

**TABLE 2:** Prescription rates of all psychotropic drug categories per 1000 person‐years and for each year after the recorded diagnosis of dementia

			Number of patients with a prescription/1000 person‐years (95% CI^a^)						
Year after dementia diagnosis	N	Total person‐ years	Any psychotropic drug^b^	Anxiolytics	Antipsychotics	Antidepressants	Hypnotics	Antiepileptics	Anti‐dementia drugs
Year 1	15687	13458	420 (409; 431)	136 (130; 142)	171 (164; 178)	165 (158; 172)	124 (118; 130)	43 (39; 47)	145 (139; 152)
Year 2	11144	9326	404 (391; 417)	121 (114; 128)	162 (154; 170)	161 (153; 169)	113 (106; 120)	44 (40; 48)	168 (160; 177)
Year 3	7635	6199	439 (423; 456)	124 (116; 133)	181 (170; 192)	183 (172; 194)	122 (114; 131)	44 (39; 50)	188 (178; 200)
Year 4	4805	3772	472 (451; 495)	132 (120; 144)	195 (181; 210)	203 (189; 218)	124 (113; 136)	50 (43; 58)	207 (192; 222)
Year 5	2827	2141	481 (452; 511)	131 (116; 147)	202 (184; 222)	214 (195; 234)	122 (108; 138)	51 (42; 61)	230 (210; 251)
Year 6	1511	1066	513 (472; 558)	124 (104; 147)	225 (198; 255)	223 (196; 253)	120 (101; 143)	53 (40; 69)	237 (210; 269)
Year 7	721	463	561 (497; 634)	151 (120; 191)	234 (193; 282)	275 (231; 328)	114 (86; 149)	62 (43; 90)	262 (219; 314)
Year 8	252	110	801 (649; 989)	244 (167; 358)	283 (197; 406)	400 (296; 541)	177 (113; 279)	89 (47; 168)	209 (136; 322)
**Total study period**	15687	36534	237 (232; 242)	83 (81; 87)	124 (120; 128)	92 (89; 95)	77 (74; 80)	24 (22; 25)	83 (80; 86)

^a^ Confidence Interval.

^b^ Including anxiolytics, antipsychotics, antidepressants, hypnotics, and antiepileptics (i.e., antidementia drugs were not included).

During the first 7 years after the recorded diagnosis, the most prescribed drugs were antidepressants, antipsychotics and antidementia drugs, followed by anxiolytics and hypnotics and lastly antiepileptics. Prescription rates of anxiolytics and hypnotics were relatively stable over time, with a strong increase in the last year(s). Antipsychotic and antidepressant prescription rates increased steadily over time, with a stronger increase in the last years. Antidementia drug prescription rates also increased steadily over time, but decreased significantly after year 7. Antiepileptic prescription rates were low and steady over time.

Tables S2–S7 show the prescription rates for subcategories (e.g., typical and atypical antipsychotics). Most anxiolytic prescriptions included benzodiazepine derivatives. Most antipsychotics prescriptions involved typical antipsychotics from the first to the sixth year after diagnosis, while in years seven and eight atypical antipsychotics were most frequently prescribed. Selective Serotonin Reuptake Inhibitors (SSRIs) were the most often prescribed antidepressants. In case of a hypnotic prescription, this was usually a benzodiazepine derivative or benzodiazepine‐related drug. Most anti‐epileptic drug prescriptions were categorized as ‘Other antiepileptics (N03AX)’. Anticholinesterases were the most commonly prescribed anti‐dementia drugs.

## 4 DISCUSSION

Among almost 16,000 people with dementia, psychotropic drug prescription rates in primary care were substantial and increased significantly during the disease trajectory. Antidepressants, antipsychotics ant anti‐dementia drugs were most often prescribed, followed by anxiolytics and hypnotics and lastly by antiepileptics.

### 4.1 Interpretation and comparison with literature

Two other large studies with representative samples examined psychotropic rates over time in community‐dwelling people with dementia.^13^, ^14^ In Finland, a prevalence of 37% at the time of diagnosis was found, increasing to 50% 4 years later among more than 70,000 persons with Alzheimer's disease. Although we estimated the prevalence of psychotropics in terms of 1000 person‐years, which is not directly comparable with a percentage, our findings appear more or less in line with these results. In contrast, our study found that most frequently prescribed were antidepressants and antipsychotics, whereas in Finland benzodiazepines and related drugs were most prevalent at the time of diagnosis (21%), followed by antidepressants (19%), and antipsychotics (9%).^14^ Four years after diagnosis, antipsychotic, and antidepressant prescription rates increased to 24% and 28% respectively, which is a steeper increase during this time period than found in our study. Differences with our results may be due to, for example, different prescription policies in countries, differences in sample characteristics (e.g., milder stages with less deterioration in our sample), or because we incorporated only prescriptions recorded in the GP's EHR (while the study from Finland included prescription drug purchases recorded in pharmacies). In the United Kingdom, antidepressant and antipsychotic prescriptions in general practice were most prevalent in more than 50,000 persons with dementia (25% and 11% respectively at the time of diagnosis) and increased to 32% and 19% 4 years later while the prevalence of hypnotics and anxiolytics was lower and remained quite stable over time.^13^ This is in line with our results.

The observed gradual increase in psychotropic drug prescription rates following dementia diagnosis in our study is expected, as the risk of developing neuropsychiatric symptoms during the progression of dementia increases. However, there are serious associated risks of psychotropics use in older persons with dementia. Clinical guidelines therefore recommend nonpharmacological treatment approaches as first‐line treatment for neuropsychiatric symptoms and advise that psychotropic drugs are only used when these approaches are unsuccessful, or when the presence of these neuropsychiatric symptoms can lead to harm for the person with dementia or the environment.^6^, ^30^ Unfortunately, our data did not provide information about the use of nonpharmacological interventions and prescription indications, so we cannot draw conclusions about the (in)appropriateness of psychotropic drug prescriptions. Contrary to the situation in institutional care where staff is trained, informal caregivers in the home care situation often do not have the skills to use nonpharmacological approaches. This may lower the threshold to prescribing psychotropic medication.

Psychotropic drug prescriptions in people with dementia have been more extensively studied in the nursing home setting, with worldwide estimates of 66%–79%.^9^, ^10^, ^11^ These rates are comparable with the prescription rates we found in the last years of our observation period. The steep increase in psychotropic prescriptions in the seventh and eighth year can probably be explained by the accumulation of problems that often occurs in the end‐stage of dementia. Previous studies have shown that the occurrence and severity of neuropsychiatric symptoms increase throughout the disease trajectory,^1^, ^2^ and in the last stage, it is likely that care providers try everything to prevent untenable situations. People with dementia in nursing homes are known to experience an overall increase in prescribing in the last stage of life,^31^, ^32^, ^33^ and high levels of medication prescription (including antidepressants, hypnotics, and antipsychotics) have also been found in primary care among dementia patients during their last year of life.^34^ The increased psychotropic prescription rates we observed during the last years of the study are in line with these previous findings. However, the numbers are low in the last follow‐up years so the results must be interpreted with caution.

Reflecting on the type of psychotropic drugs that were prescribed, typical antipsychotics were most frequently prescribed in the first 6 years after the dementia diagnosis was recorded, while atypical antipsychotics were most often prescribed in the seventh and eighth year after diagnosis. Dutch GPs usually have less experience with atypical antipsychotics and mainly prescribe typical antipsychotics.^35^ In order to reduce the risk of neuroleptic‐induced parkinsonism, typical antipsychotics are usually prescribed for short periods of time, while atypical antipsychotics are more often used for continuous maintenance antipsychotic treatment. The shift from typical to atypical antipsychotics in the later years after dementia diagnosis is possibly due to an increase in the severity of neuropsychiatric symptoms and the felt need for maintained antipsychotic treatment.

SSRIs were the most prevalent prescribed antidepressants during the disease trajectory. This is in line with the recommendations in Dutch guidelines. Depression guidelines for Dutch GPs advise to start antidepressant treatment in older people with an SSRI.^36^, ^37^ In addition, multiple Dutch guidelines for the treatment of people with dementia addressed the prescription of SSRIs for behavioral problems.^30^, ^38^, ^39^

Most anxiolytic prescriptions involved benzodiazepine derivatives. In case of hypnotics, this was usually a benzodiazepine derivative or benzodiazepine‐related drug. The Dutch guideline recommends considering benzodiazepines for people with dementia only in case of severe (pathological) anxiety and stress when psychosocial interventions have not been successful, and recommend a short‐acting benzodiazepine without active metabolites for a maximum period of four weeks. Although we did not examine the duration of prescriptions, the stable trend in benzodiazepines prescriptions seems to imply that these medications are frequently prescribed for long‐term use.

Anti‐epileptic prescription rates were low and steady over time, and most of the prescriptions involved “other antiepileptics (N03AX)”, which are usually prescribed for epilepsy. However, the majority of people with an antiepileptic prescription did not have a recorded epilepsy diagnosis in the EHR during the study period (data not shown). This could indicate that these medications were prescribed for other reasons, or that epilepsy diagnoses were under recorded in the medical record.

Antidementia drug prescription rates, mainly anticholinesterases, steadily increased during the disease trajectory, but decreased in the last follow‐up year. As dementia medication is usually prescribed for persons in the mild to moderate stages, the decreased rate in the last year probably indicates that the remaining sample of patients had more severe dementia. The Dutch dementia guideline for GPs advises against prescribing antidementia medication as a standard practice, because of the limited expected benefits. If patients desire a pilot treatment or in case of disturbing neuropsychiatric symptoms, GPs are advised to refer to a medical specialist with experience in prescribing these medications^30^ and whose guidelines feature prescribing anti‐dementia drugs more prominently.^38^

### 4.2 Strengths and limitations

To our knowledge, this is one of the few studies to examine psychotropic drug prescription rates in primary care among people with dementia during the disease trajectory using large, nationally representative, routinely recorded data from a large number of general practices. We were able to describe the development of drug prescription during the disease trajectory. The use of routinely recorded data also overcomes important problems such as selective drop out, recall errors, and self‐report bias.^40^, ^41^

A limitation of this study is the lack of information about the severity and type of dementia, rate of decline, and neuropsychiatric symptoms, which are not (yet) structurally recorded in a standardized way in GPs EHRs. We could therefore not relate the prescription of psychotropic drugs to the stage of dementia and to possible neuropsychiatric symptoms for which they may have been prescribed. In addition, we did not have information about the indications of prescriptions and use of nonpharmacological interventions before psychotropic drugs were prescribed, which is important to gain insight into the (in)appropriateness of prescribing. Although we used data from an 8‐year period, the sample's median follow‐up time of 2 years was rather short, partly because people were included when a dementia diagnosis was recorded between 2008 and 2016 and could therefore reach the end of the data extraction period relatively quickly.

Furthermore, dementia is known to be poorly recognized in primary care, and GPs are reluctant to record the diagnosis.^42^, ^43^, ^44^ Underreporting of dementia diagnoses in GP records occurs especially in the early stages. The diagnosis is usually made by specialists in memory or other specialized outpatient clinics and then communicated to the GP, which may lead to a registration delay. For these reasons, the prescription rates in this study are perhaps less representative for people in the early stages of dementia. Lastly, the analysis of prescriptions rather than dispensations, which will be more closely related to which drugs people with dementia were actually using, can be seen as a limitation. Previous research has shown that on average people with Alzheimer's disease achieved 84% medication adherence, with 70% scoring 80% or higher.^45^, ^46^ Although this indicates an acceptable range of adherence for the majority of people with Alzheimer's disease, there was considerable variability in adherence, ranging from 17% to 100%, and no specific information was provided about adherence to psychotropic medications. We therefore emphasized throughout the paper that all rates involve prescriptions and not actual use of psychotropics.

## 5 CONCLUSIONS AND IMPLICATIONS

In the years after a dementia diagnosis is recorded in the GPs EHR, the prevalence of psychotropic drugs prescriptions is substantial and increases steadily among persons with dementia. Future studies are needed to give a better insight into the reasons for the rather high prevalences, the inappropriateness of the prescribed drugs, and its related factors. The first look at psychotropic prescription rates over time provided by this study may stimulate GPs to (re)consider their prescription policy, consider risks and benefits more carefully and examine how the need for psychotropic medication can be reduced with better support of informal caregivers.

## AUTHOR CONTRIBUTIONS

All authors were involved in designing the study. Karlijn J. Joling acquired the data. Maud ten Koppel and Karlijn J. Joling performed the data analysis and wrote the first draft of the manuscript. Jos W.R. Twisk provided statistical advice. All authors interpreted the data, critically revised the manuscript, read and approved the manuscript.

## CONFLICT OF INTEREST

The authors declare that they have no conflict of interest.

## Supporting information

Supplementary Material

## Data Availability

Research data are not shared as these are stored in the safe environment of Statistics Netherlands and not allowed to leave this environment.
