# Supplementary material for: Psychotropic drug prescription rates in primary care for people with dementia from recorded diagnosis onwards
Source: Int J Geriatr Psychiatry. 2020 Oct 16;36(3):443–51. doi: 10.1002/gps.5442 (PMC7894336; doi:10.1002/gps.5442)
Supplement: Supplementary file 1 — Supplementary Material [file GPS-36-443-s001.docx]

**APPENDIX**

**Table 1:** Categories of psychotropic drugs

| **Main categories** | **Subcategories (ATC-5 code)**† | | | |
| --- | --- | --- | --- | --- |
| Anxiolytics | Benzodiazepine derivatives (N05BA) | Azaspirodecanedione derivatives (N05BE) (buspirone) | Anxiolytics, other (N05BB; N05BC; N05BD; N05BX) |  |
| Antipsychotics | Atypical antipsychotics (N05AE; N05AH; N05AX) | Typical antipsychotics (N05AB; N05AC; N05AD; N05AF; N05AG; N05AL) | Antipsychotics, other (N05AA) |  |
| Antidepressants | TCA‡ (N06AA) | SSRI $ (N06AB) | Antidepressants, other (N06AF; N06AG; N06AX) |  |
| Hypnotics | Benzodiazepine derivates + benzodiazepine related drugs (N05CD; N05CF) | Melatonin receptor agonists (N05CH ) | Hypnotics, other (N05CA; N05CB; N05CC; N05CE; N05CM; N05CX) |  |
| Antiepileptics | Carboxamide derivatives (N03AF, e.g. N03AF01 carbamazepine) | Fatty acid derivatives (N03AG, e.g. N03AG01 valproic acid) | Other antiepileptics (N03AX) | Anti-epileptics, other (N03AA; N03AB; N03AC; N03AD; N03AE) |
| Anti-dementia drugs | Anticholinesterases (N06DA) | Anti-dementia drugs, other (N06DX) |  |  |

† Anatomical Therapeutic Chemical (ATC) Classification System

‡ Tricyclic antidepressants

$ Selective serotonin reuptake inhibitor

**Table 2:** Prescription rates of anxiolytics per 1000 person years after the diagnosis of dementia

|  | **Number of patients with a prescription per 1000 person years (95% CI**†**)** | | | | |
| --- | --- | --- | --- | --- | --- |
| **Year after dementia diagnosis** | Any anxiolytic | Benzodiazepine derivatives | Azaspirodecanedione derivatives | Anxiolytics, other |  |
| Year 1 | 136 (130; 142) | 132 (126; 139) | 0 (0;1) | 4 (3; 5) |  |
| Year 2 | 121 (114; 128) | 117 (110; 124) | NA‡ | 5 (3; 6) |  |
| Year 3 | 124 (116; 133) | 121 (113; 130) | NA‡ | 3 (2; 5) |  |
| Year 4 | 132 (120; 144) | 127 (116; 139) | NA‡ | 4 (2; 7) |  |
| Year 5 | 131 (116; 147) | 129 (115; 146) | NA‡ | 2 (1; 6) |  |
| Year 6 | 124 (104; 147) | 120 (101; 143) | NA‡ | 2 (1; 9) |  |
| Year 7 | 151 (120; 191) | 151 (120; 191) | NA‡ | NA‡ |  |
| Year 8 | 244 (167; 358) | 244 (167; 358) | NA‡ | NA‡ |  |
| **Total study period** | 83 (81; 87) | 81 (78; 84) | 0 (0.1; 0) | 3 (2; 3) |  |

† Confidence Interval.

‡ not applicable due to very small numbers

**Table 3:** Prescription rates of antipsychotics per 1000 person years after the diagnosis of dementia

|  | **Number of patients with a prescription per 1000 person years (95% CI**†**)** | | | | |
| --- | --- | --- | --- | --- | --- |
| **Year after dementia diagnosis** | Any antipsychotic | Atypical antipsychotics | Typical antipsychotics | Antipsychotics, other |  |
| Year 1 | 171 (164; 178) | 65 (61; 70) | 117 (111; 123) | 3 (2; 4) |  |
| Year 2 | 162 (154; 170) | 64 (59; 69) | 106 (99; 113) | 3 (2; 4) |  |
| Year 3 | 181 (170; 192) | 68 (62; 75) | 119 (111; 129) | 4 (3; 6) |  |
| Year 4 | 195 (181; 210) | 76 (67; 85) | 126 (115; 138) | 4 (3; 7) |  |
| Year 5 | 202 (184; 222) | 84 (72; 98) | 126 (112; 142) | 7 (4; 11) |  |
| Year 6 | 225 (198; 255) | 94 (77; 115) | 134 (113; 159) | 9 (5; 17) |  |
| Year 7 | 234 (193; 282) | 129 (100; 167) | 118 (90; 154) | 13 (5; 29) |  |
| Year 8 | 283 (197; 406) | 187 (120; 291) | 85 (38; 189) | 17 (4; 75) |  |
| **Total study period** | 124 (120; 128) | 45 (43; 48) | 92 (89; 95) | 2 (2; 3) |  |

† Confidence Interval.

‡ not applicable due to very small numbers

**Table 4:** Prescription rates of antidepressants per 1000 person years after the diagnosis of dementia

|  | **Number of patients with a prescription per 1000 person years (95% CI**†**)** | | | |
| --- | --- | --- | --- | --- |
| **Year after dementia diagnosis** | Any antidepressant | TCA | SSRI | Other antidepressants |
| Year 1 | 165 (158; 172) | 35 (32; 38) | 88 (83; 93) | 53 (49; 57) |
| Year 2 | 161 (153; 169) | 31 (28; 35) | 86 (80; 92) | 51 (46; 56) |
| Year 3 | 183 (172; 194) | 35 (30; 40) | 99 (91; 107) | 60 (54; 66) |
| Year 4 | 203 (189; 218) | 38 (32; 45) | 105 (95; 116) | 68 (60; 77) |
| Year 5 | 214 (195; 234) | 39 (32; 49) | 113 (99; 128) | 68 (58; 80) |
| Year 6 | 223 (196; 253) | 38 (28; 52) | 119 (100; 142) | 74 (59; 93) |
| Year 7 | 275 (231; 328) | 52 (34; 77) | 143 (111; 183) | 86 (62; 117) |
| Year 8 | 400 (296; 541) | 117 (67; 203) | 163 (101; 265) | 128 (76; 216) |
| **Total study period** | 92 (89; 95) | 19 (18; 21) | 50 (48; 52) | 31 (29; 33) |

† Confidence Interval.

‡ not applicable due to very small numbers

**Table 5:** Prescription rates of hypnotics per 1000 person years after the diagnosis of dementia

|  | **Number of patients with a prescription per 1000 person years (95% CI**†**)** | | | |
| --- | --- | --- | --- | --- |
| **Year after dementia diagnosis** | Any hypnotic | Benzodiazepine derivates and  benzodiazepine related drugs | Melatonin receptor agonists | Hypnotics, other |
| Year 1 | 124 (118; 130) | 117 (112; 123) | 8 (7; 10) | 2 (1; 3) |
| Year 2 | 113 (106; 120) | 107 (101; 114) | 6 (5; 8) | 1 (1; 3) |
| Year 3 | 122 (114; 131) | 115 (107; 124) | 8 (6; 10) | 1 (0; 3) |
| Year 4 | 124 (113; 136) | 117 (107; 129) | 8 (6; 12) | 1 (1; 4) |
| Year 5 | 122 (108; 138) | 114 (100; 129) | 9 (5; 14) | 2 (1; 5) |
| Year 6 | 120 (101; 143) | 112 (93; 134) | 9 (5; 17) | NA‡ |
| Year 7 | 114 (86; 149) | 98 (73; 131) | 14 (6; 32) | NA‡ |
| Year 8 | 177 (113; 279) | 152 (94; 247) | 33 (11; 98) | NA‡ |
| **Total study period** | 77 (74; 80) | 72 (70; 75) | 6 (5; 7) | 1 (1; 2) |

† Confidence Interval.

‡ not applicable due to very small numbers

**Table 6:** Prescription rates of antiepileptic drugs per 1000 person years after the diagnosis of dementia

|  | **Number of patients with a prescription per 1000 person years (95% CI**†**)** | | | | | |
| --- | --- | --- | --- | --- | --- | --- |
| **Year after dementia diagnosis** | Any antiepileptic | Carboxamide derivatives | Fatty acid derivatives | Other antiepileptics (N03AX) | Anti-epileptics, other (N03AA; N03AB; N03AC; N03AD; N03AE) |  |
| Year 1 | 43 (39; 47) | 6 (5; 8) | 9 (8; 11) | 24 (21; 27) | 10 (8; 12) |  |
| Year 2 | 44 (40; 48) | 7 (6; 9) | 8 (6; 10) | 24 (21; 28) | 10 (8; 13) |  |
| Year 3 | 44 (39; 50) | 6 (5; 9) | 8 (6; 11) | 22 (19; 26) | 12 (10; 16) |  |
| Year 4 | 50 (43; 58) | 7 (5; 10) | 10 (7; 14) | 25 (20; 31) | 14 (10; 18) |  |
| Year 5 | 51 (42; 61) | 6 (3; 10) | 10 (6; 16) | 23 (17; 31) | 14 (9; 19) |  |
| Year 6 | 53 (40; 69) | 7 (4; 15) | 12 (7; 21) | 25 (17; 37) | 11 (6; 19) |  |
| Year 7 | 62 (43; 90) | NA‡ | 11 (4; 28) | 40 (25; 64) | 10 (4; 26) |  |
| Year 8 | 89 (47; 168) | NA‡ | NA‡ | 32 (10; 98) | 18 (4; 86) |  |
| **Total study period** | 24 (22; 25) | 3 (3; 4) | 5 (4; 6) | 13 (12; 14) | 6 (5; 7) |  |

† Confidence Interval.

‡ not applicable due to very small numbers

**Table 7:** Prescription rates of anti-dementia drugs per 1000 person years after the diagnosis of dementia

|  | **Number of patients with a prescription per 1000 person years (95% CI**†**)** | | |
| --- | --- | --- | --- |
| **Year after dementia diagnosis** | Any anti-dementia drug | Anticholinesterases | Other anti-dementia drugs |
| Year 1 | 145 (139; 152) | 128 (122; 135) | 23 (21; 26) |
| Year 2 | 168 (160; 177) | 148 (140; 156) | 30 (27; 34) |
| Year 3 | 188 (178; 200) | 164 (154; 175) | 40 (35; 45) |
| Year 4 | 207 (192; 222) | 179 (166; 193) | 37 (32; 43) |
| Year 5 | 230 (210; 251) | 193 (175; 213) | 59 (49; 71) |
| Year 6 | 237 (210; 269) | 204 (178; 233) | 65 (51; 83) |
| Year 7 | 262 (219; 314) | 223 (183; 271) | 71 (50; 100) |
| Year 8 | 209 (136; 322) | 199 (129; 308) | 32 (9; 119) |
| **Total study period** | 83 (80; 86) | 72 (69; 75) | 18 (16; 19) |

† Confidence Interval.
